# Retrospective one-million-subject fixed-cohort survey of utilization of emergency departments due to traumatic causes in Taiwan, 2001–2010

**DOI:** 10.1186/s13017-016-0098-x

**Published:** 2016-08-30

**Authors:** Nan-Ping Yang, Dinh-Van Phan, Yi-Hui Lee, Jin-Chyr Hsu, Ren-Hao Pan, Chien-Lung Chan, Nien-Tzu Chang, Dachen Chu

**Affiliations:** 1 Department of Surgery & Orthopedics, Keelung Hospital, Ministry of Health & Welfare, Keelung, Taiwan; 2 Faculty of Medicine, School of Medicine, National Yang-Ming University, Taipei, Taiwan; 3 Institute of Public Health and Community Medicine Research Center, National Yang-Ming University, Taipei, Taiwan; 4 Department of Information Management, Yuan Ze University, Taoyuan, Taiwan; 5 Innovation Center for Big Data and Digital Convergence, Yuan Ze University, Taoyuan, Taiwan; 6 Department of Nursing, School of Nursing, College of Medicine, Chang-Gang University, Taoyuan, Taiwan; 7 Department of Medicine, Taipei Hospital, Ministry of Health & Welfare, Taipei, Taiwan; 8 School of Nursing, College of Medicine, National Taiwan University, Taipei, Taiwan; 9 Department of Neurosurgery, Taipei City Hospital, Taipei, Taiwan; 10 Department of Health Care Management, National Taipei University of Nursing and Health Sciences, Taipei, Taiwan

**Keywords:** Emergency department, Trauma, Utilization

## Abstract

**Background:**

Epidemiological study was needed to evaluate trends in emergency department (ED) utilization that could be taken into account when making policy decisions regarding the delivery and distribution of medical resources.

**Methods:**

A retrospective fixed-cohort study of emergency medical utilization from 2001 to 2010 was performed based on one-million people sampled in 2010 in Taiwan. Focusing on traumatic cases, the annual incidences in various groups split according to sex and age were calculated, and further information regarding location of trauma and type of trauma was obtained.

**Results:**

In 2010, significantly greater proportions of male and younger subjects were visitors to EDs with a traumatic injury. During 2001–2010, the number of both traumatic cases and non-traumatic cases presenting at EDs significantly increased (average annual percentage change, AAPC 4.7 and 3.6, respectively) and a significantly greater direct medical cost associated with traumatic cases than non-traumatic cases was noted. Focusing on traumatic cases, most of these cases were directed to highest-level hospitals, accounting for 73.5–78.8 % of all traumatic cases, with a significant AAPC of 5.6. The traumatic ED visit annual incidence in males was 58.63 in 2001, which significantly increased to 69.35 per 1000 persons in 2010 (AAPC 1.5); and in females was 38.96 in 2001, which significantly increased to 50.73 per 1000 persons in 2010 (AAPC 2.5). Most of the traumatic cases treated in EDs were minor injuries, such as contusion with the skin intact, open wound of the upper limbs, open wound of the head, neck, or trunk, and other superficial injury (accounting for about 60 % of all cases). The traumatic categories of sprains/strains of joints and adjacent muscles, fractures of upper limbs, fractures of lower limbs, and fractures of the spine/trunk required greater medical resources and significantly positive AAPC values (4.3, 4.0, 4.5 and 6.8, respectively).

**Conclusions:**

Increased ED utilization due to traumatic causes, as assessed by the annual number of cases and incidence, average direct medical cost and highest-level hospital utilization, was observed from 2001 to 2010. Orthopedic-related injuries, including soft tissue trauma of extremities and various fractures, were the categories with the greatest increase in incidence.

## Background

Globally, injury is a major cause of death and disability. The burden of injury is greatest in developing countries, and trauma registries are known to be integral in the monitoring and improvement of trauma care [1]. There remains great international variability in terms of patient mix, process of care, and performance of different pre-hospital trauma care systems [2]. To date, the classification, measurement and improvement of data quality in trauma registries have been inconsistent [3]. There is no doubt that injury is a large issue for the health care system, and in particular for emergency management units.

Usually, patients suffering a traumatic injury and other acute diseases constitute the majority of ambulance transports, and adult patients aged 15–60 years are the principal users of Emergency Medical Services (EMSs) [4]. Furthermore, triage, inter-hospital transfer processes and protocols for inter-hospital transfer are also central to trauma systems [5, 6]. From the viewpoints of public health and health policy, epidemiological study is needed in order to evaluate trends in health care utilization, which could inform policy decision-making for the redistribution of medical resources, especially in developing countries.

## Methods

### Data source, security, and quality control

Taiwan launched a single-payer National Health Insurance (NHI) program on March 1, 1995. As of 2014, 99.9 % of Taiwan’s population was enrolled [7]. Foreigners in Taiwan are also eligible for this program. The database of this program contains registration files and original claim data for reimbursement. Large computerized databases derived from this system by the National Health Insurance Administration (the former Bureau of National Health Insurance, BNHI), Ministry of Health and Welfare (the former Department of Health, DOH), Taiwan and maintained by the National Health Research Institutes (NHRI), Taiwan, are provided to scientists in Taiwan for research purposes. Only citizens of the Republic of China who fulfill the requirements of conducting research projects are eligible to apply for the National Health Insurance Research Database (NHIRD). The NHIRD includes nationwide population-based data with good quality control and representation and is provided to scientists in Taiwan for research purposes [8–12].

### Ethics approval, and consent for participation or publication

The study design was a retrospective health data analytic study. The protocol was evaluated and approved by the Institutional Review Board (IRB) of Taipei Hospital, Ministry of Health & Welfare, Taiwan, which has been certificated by the Ministry of Health and Welfare, Taiwan (IRB Approval Number: TH-IRB-0015-0003).

The information related to all subjects is encrypted using a double scrambling protocol for research purposes to protect the privacy of patients. Theoretically, it is impossible to query the data alone to identify individuals at any level using this database. No any individual informed consent of all the insured is requested. All researchers who wish to use the NHIRD are required to sign a written agreement declaring that they have no intention of attempting to obtain information that could potentially violate the privacy of patients or care providers. The use of NHIRD is limited to research purposes only. Applicants must follow the Computer-Processed Personal Data Protection Law and related regulations of National Health Insurance Administration and NHRI, and an agreement must be signed by the applicant and his/her supervisor upon application submission.

The application of the present study was reviewed by the NHI Administration, who gave their agreement to the planned analysis of the NHIRD and released the data. (Data approval Number: NHIRD-104-183).

### Source and definition of study population

**Fig. 1:**
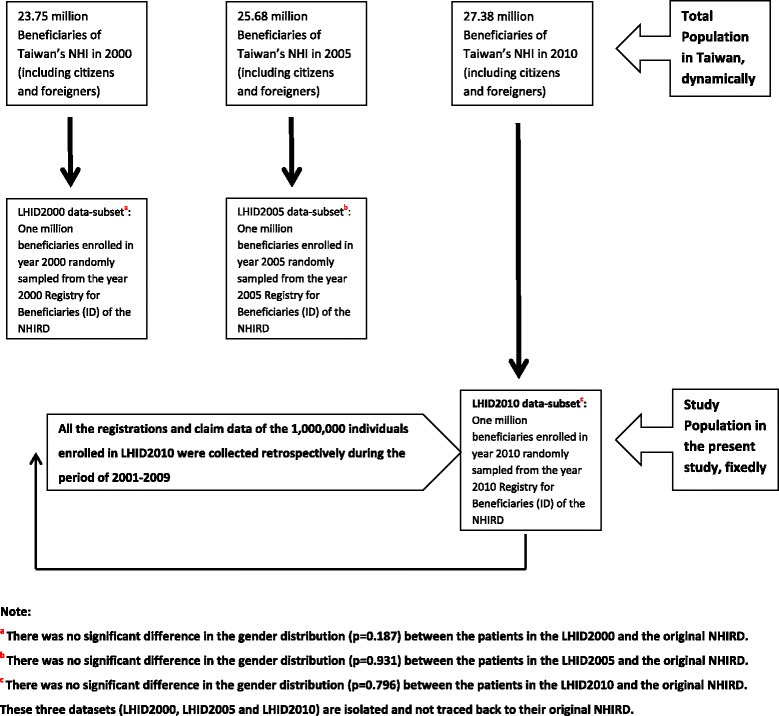
The relationship of study population and total population in the present study

In this retrospective fixed-cohort study, the records of 1 million people in the NHIRD sampled in 2010 were used to perform an epidemiological and descriptive study of emergency medical utilization in Taiwan from 2001 to 2010. This specific longitudinal health insurance dataset (LHID) is the latest cohort data in Taiwan that was named LHID2010 (see Fig. 1) [13]. LHID2010, used as the study population in the present study, contains the entire original claim data of 1,000,000 beneficiaries enrolled in year 2010 randomly sampled from the year 2010 Registry for Beneficiaries (ID) of the NHIRD, where registration data of everyone who was a beneficiary of the National Health Insurance program during the period of Jan. 1st 2010 to Dec. 31 2010 were drawn for random sampling. There are approximately 27.38 million individuals in this registry. All the registration and claim data of these 1,000,000 individuals collected by the National Health Insurance program constitute the LHID2010. There was no significant difference in the gender distribution (*p*-value = 0.796) between the patients in the LHID2010 and the original NHIRD [13].

The entire medical claim data of the patients enrolled in LHID2010 was collected in the year 2010 and during the period of 2001–2009 retrospectively. All incident ED visits of the 1,000,000 studied subjects in each calendar year were evaluated. The causes of ED visits were divided into 2 groups: traumatic (ICD-9-CM diagnostic codes ranging from 800.X to 959.X) and non-traumatic. Traumatic cases were defined as subjects who visited an ED due to a trauma event once or more in 1 year, while the non-traumatic cases were defined as subjects who visited an ED for reasons entirely due to a non-traumatic event. Furthermore, each ED visit of the subjects was analyzed to assess the number of annual ED visits and the direct medical cost per ED case. Focusing on traumatic ED cases, the distribution of hospital level, annual incidence by gender, annual incidence by age strata, and detailed information regarding traumatic location/type (classified into 20 categories) [11] were studied.

### Statistical analysis

Descriptive statistics are represented by numbers of cases, percentages, and means with standard deviation (SD). The chi-square test or independent *t*-test was used to analyze significant differences between groups for categorical or continuous variables, respectively. The average annual percentage change (AAPC) and its 95 % confidence interval (CI) was calculated using the Join-point Regression Program Ver.4.2.0.2 in order to test for time trends. 95 % CI of cumulative incidence (ci) cab be calculated as the followed formula: ci +/− 1.96√ci(1-ci)/N. All analyses of data were conducted using the Hadoop big data distributed computing environment and the Impala massive parallel processing (MPP) SQL query engine (Cloudera Corporation), and statistical calculations were performed using the Statistical Package for Social Sciences for Windows (SPSS Ver.22.0).

## Results

Of the enrolled 1 million people sampled in 2010, 176,126 subjects visited an ED, who were classified into 59,900 traumatic subjects (34 %) and 116,226 non-traumatic subjects (66 %). Significantly greater proportions of male subjects (57 %) and younger patients (64 %) presented as traumatic ED cases (see Table 1).

**Table 1:** Retrospective one-million-subject fixed-cohort survey of utilization of emergency departments (EDs) due to traumatic or non-traumatic causes in Taiwan, 2010

Age (years)/Gender	Traumatic subjects		Non-traumatic subjects			ED-utilized subjects in 2010		One million study subjects based on LHID2010^a^	
	N	(%)	N	(%)	*p* value of *χ* ^2^ test	N	(%)	N (%)	
Total subjects who visited the ED	59,900	34.01	116,226	65.99		176,126	100.00	1,000,000	100
Age (years)									
0–29	25,801	43.07	46,062	39.63	.000	71,863	40.80	372,341	37.23
30–44	12,286	20.51	22,493	19.35		34,779	19.75	251,103	25.11
45–59	11,047	18.44	21,024	18.09		32,071	18.21	223,100	22.31
60+	10,766	17.97	26,647	22.93		37,413	21.24	153,456	15.35
Gender									
Female	25,750	42.99	60,968	52.46	.000	86,718	49.24	507,577	50.76
Male	34,150	57.01	55,258	47.54		89,408	50.76	492,423	49.24

^a^LHID2010: longitudinal health insurance dataset randomly selected in 2010

All ED visits of the subjects were analyzed as individual cases, and the annual number of ED visits, average direct ED medical cost per case, and time trends were evaluated, as shown in Table 2. During 2001–2010 in Taiwan, the numbers of both traumatic cases and non-traumatic cases sent to EDs significantly increased (average annual increase ratio 4.7 % (95 % CI: 3.2–6.2 %) and 3.6 % (95 % CI: 1.0–6.4 %), respectively). Although fewer traumatic cases were seen in EDs than non-traumatic cases, the direct medical cost of traumatic cases was significantly greater than that of non-traumatic cases, and a significant increasing trend of the direct medical cost was observed for both types of case. Focusing on traumatic cases, most (i.e., 73.5–78.8 % of all traumatic cases) were taken to highest-level hospitals, this incidence exhibiting a significant increasing annual trend of 5.6 % during 2001–2010 in Taiwan.

**Table 2:** Trends and comparisons of medical utilization in traumatic and non-traumatic ED cases in Taiwan, 2001–2010

	2001–2002	2003–2004	2005–2006	2007–2008	2009–2010	AAPC^a^	95 % CI^b^ of AAPC
Annual ED visits, case numbers							
Traumatic cases	63,477	71,221	76,904	80,703	94,176	4.7	3.2, 6.2
Non-traumatic cases	127,988	133,837	141,391	145,217	175,571	3.6	1.0, 6.4
Direct medical cost (US$), per visit							
Traumatic cases, mean (SD)	53.51 (97.20)	60.98 (72.12)	67.86 (86.65)	72.08 (84.97)	78.61 (100.81)	4.8	3.6, 6.0
Non-traumatic cases, mean (SD)	40.53 (94.04)	46.37 (113.32)	56.11 (104.56)	65.24 (110.76)	75.49 (116.98)	8.2	7.5, 9.0
p value for *t* test	0.000	0.000	0.000	0.000	0.000		
Level of hospital visited by traumatic cases, per year							
Medical center, No. (%)	46,647 (73.49)	52,296 (73.43)	57,909 (73.91)	61,737 (76.50)	74,179 (78.77)	5.6	4.0, 7.3
Regional hospital, No. (%)	15,770 (24.84)	17,557 (24.65)	18,590 (23.73)	17,385 (21.54)	18,513 (19.66)	1.6	−1.0, 4.2
Local hospital or other, No. (%)	1061 (01.67)	1369 (01.92)	1852 (02.36)	1581 (01.96)	1484 (01.58)	4.2	−5.1, 14.3

Currency: USD: NTD = 1: 32

^a^ *AAPC* average annual percentage change; the AAPC was significantly different from zero at alpha = 0.05

^b^ *95 % CI* 95 % confidence interval

**Fig. 2:**
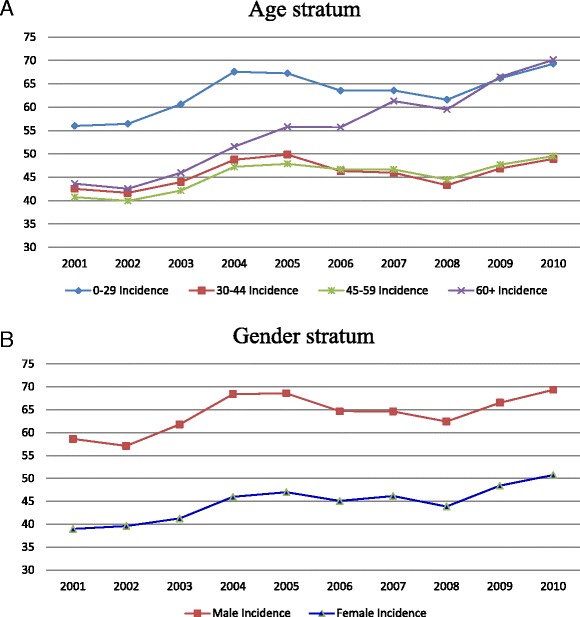
**a**, **b** Linear trends of the annual incidence of traumatic ED visiting subjects in various subgroups

Furthermore, still focusing on traumatic cases, the annual ED visit incidence and the annual trend were analyzed by gender and age. The point estimation of incidence was calculated by the number of subjects visiting ED due to traumatic causes divided by the numbers of various gender and age stratums among the one million study population in each calendar year. Because the study population of 1,000,000 subjects was randomly sampled in 2010 whose information could not be traced back to their original NHIRD, point estimates of cumulative incidences were calculated and their 95 % CIs could be predicted only in 2010. Even though the same fixed one-million subjects were studied during 2001–2009, all the 95 % CIs of point estimates of incidence were not projected accounting for actually dynamic total population rather than an assumedly fixed total population in Taiwan for the past 10 years. As shown in Table 3, the annual incidence of traumatic ED visitors in male subjects was 58.63 per 1000 persons, significantly increasing to 69.35 (95 % CI: 68.85–69.85) per 1000 persons from 2001 to 2010, with an average annual change of 1.5 % (95 % CI: 0.1–2.8 %); that in the female subjects was 38.96 per 1000 persons, significantly increasing to 50.73 (95 % CI: 50.30–51.16) per 1000 persons from 2001 to 2010, with an average annual change of 2.5 % (95 % CI: 1.3–3.7 %). Analyzing the subjects by age, significant increasing trends in the annual incidence of traumatic ED visitors were noted in subjects aged 29 years or under and ones aged 60 years or order. However, as illustrated in Fig. 2, a dramatically increasing linear trend of 43.61/1000 to 70.16/1000 during 2001–2010 was noted in the subject population aged 60 years or older, with an AAPC of 5.7 (95 % CI: 4.7–6.8), which was greater than that in other age strata groups.

**Table 3:** Characteristics of traumatic ED visitors in terms of annual incidence* of the fixed one-million subjects in Taiwan, 2001–2010 retrospectively

	2001	2002	2003	2004	2005	2006	2007	2008	2009	2010		AAPC	95 % CI of AAPC
	Inc.	Inc.	Inc.	Inc.	Inc.	Inc.	Inc.	Inc.	Inc.	Inc.	95 % CI of Inc. in 2010		
Gender													
Male	58.63	57.09	61.80	68.42	68.59	64.68	64.63	62.44	66.52	69.35	68.85–69.85	1.5	0.1,2.8
Female	38.96	39.62	41.25	45.98	47.00	45.09	46.17	43.87	48.40	50.73	50.30–51.16	2.5	1.3,3.7
Age (years)													
0–29	55.99	56.45	60.65	67.59	67.25	63.54	63.58	61.61	66.20	69.29	68.80–69.79	2.8	0.3,5.3
30–44	42.52	41.65	43.99	48.75	49.84	46.32	45.96	43.29	46.88	48.93	48.51–49.35	2.0	−3.1,7.4
45–59	40.76	39.94	42.15	47.23	47.86	46.69	46.66	44.52	47.69	49.52	49.09–49.94	2.0	0.7,3.2
60 +	43.61	42.56	45.97	51.55	55.80	55.71	61.31	59.52	66.52	70.16	69.66–70.66	5.7	4.7, 6.8

*Incidence (Inc.): cumulative incidence, 1/1000

Based on the ICD-9-CM diagnostic codes, the trauma locations of the traumatic cases in the present study were classified into 20 categories. Table 4 shows that most of the traumatic patients in EDs were treated for minor injuries, such as contusion with the skin intact, open wound of upper limb, open wound of the head, neck, or trunk, and other superficial injury (accounting for about 60 % of all cases, ranging from 58.5 to 63.3 %). Besides the above-listed and some unspecified injuries, the traumatic categories of sprains/strains of joints and adjacent muscles, fractures of upper limbs, fractures of lower limbs, and fractures of the spine/trunk required greater medical resources and significantly positive AAPC values (4.3, 4.0, 4.5 and 6.8, respectively).

**Table 4:** Distributions of traumatic categories in traumatic ED cases in Taiwan, 2001–2010

Traumatic category*	ICD-9-CM code	2001–2002	2003–2004	2005–2006	2007–2008	2009–2010	AAPC	95 % CI of AAPC
		No. (%)	No. (%)	No. (%)	No. (%)	No. (%)		
1. Fracture of skull, intracranial injury	800–804 & 850–854	11,060 (11.66)	10,229 (9.77)	8650 (7.60)	7429 (6.47)	7688 (6.09)	−5.5	−8.0, −2.1
2. Fracture of spine and trunk	805–809	870 (0.92)	835 (0.80)	989 (0.87)	1133 (0.99)	1439 (1.14)	6.8	2.2, 11.6
3. Fracture of upper limb	810–819	3058 (3.22)	3305 (3.16)	3539 (3.11)	3819 (3.33)	4193 (3.32)	4.0	3.5, 4.4
4. Fracture of lower limb	820–829	2376 (2.51)	2511 (2.40)	2597 (2.28)	2875 (2.51)	3432 (2.72)	4.5	1.8, 7.2
5. Dislocation	830–839	963 (1.02)	821 (0.78)	846 (0.74)	938 (0.82)	1037 (0.82)	1.4	−3.5, 6.5
6. Sprains and strains of joints and adjacent muscles	840–848	3472 (3.66)	3998 (3.82)	4441 (3.90)	4482 (3.91)	4975 (3.94)	4.3	2.2, 6.3
7. Internal injury of chest	860–862	151 (0.16)	139 (0.13)	128 (0.11)	090 (0.08)	132 (0.10)	−3.5	−12.3, 6.2
8. Internal injury of abdomen and pelvis	863–869	158 (0.17)	144 (0.14)	137 (0.12)	133 (0.12)	153 (0.12)	−0.7	−4.6, 3.3
9. Open wound of head, neck and trunk	870–879	15,579 (16.43)	15,939 (15.22)	15,739 (13.83)	15,062 (13.12)	14,221 (11.26)	−1.2	−2.7, 0.3
10. Open wound of upper limb	880–887	14,278 (15.06)	15,498 (14.80)	16,147 (14.19)	15,072 (13.13)	15,768 (12.48)	0.9	−1.4, 3.2
11. Open wound of lower limb	890–897	7437 (7.84)	7506 (7.17)	7932 (6.97)	7853 (6.84)	8149 (6.45)	1.1	0.4, 1.9
12. Injury to blood vessels	900–904	660 (0.70)	944 (0.90)	671 (0.59)	232 (0.20)	194 (0.15)	−17.5	−33.1, 1.7
13. Late effects of injuries and other external causes	905–909	101 (0.11)	84 (0.08)	75 (0.07)	70 (0.06)	96 (0.08)	−1.4	−9.6, 7.6
14. Superficial injury	910–919	7429 (7.83)	8580 (8.19)	10,376 (9.12)	11,497 (10.02)	13,376 (10.59)	7.6	6.4, 8.8
15. Contusion with intact skin surface	920–924	18,229 (19.22)	23,468 (22.41)	28,704 (25.23)	30,963 (26.98)	36,358 (28.78)	8.6	5.6, 11.8
16. Crush injuries	925–929	1958 (2.06)	2190 (2.09)	2237 (1.97)	2070 (1.80)	2089 (1.65)	0.4	−2.6, 3.4
17. Effects of foreign body entering through orifice	930–939	2359 (2.49)	2661 (2.54)	2844 (2.50)	2794 (2.43)	3023 (2.39)	2.8	0.7, 4.9
18. Burns	940–949	3411 (3.60)	3369 (3.22)	3320 (2.92)	3226 (2.81)	3579 (2.83)	0.3	−1.9, 2.5
19. Injury to nerves and spinal cord	950–957	107 (0.11)	155 (0.15)	163 (0.14)	179 (0.16)	227 (0.18)	8.6	3.6, 13.8
20. Certain traumatic complications and unspecified injuries	958–959	1181 (1.25)	2343 (2.24)	4246 (3.73)	4850 (4.23)	6203 (4.91)	22.4	9.6, 36.7

*Accounting for the 1st main diagnostic code only

## Discussion

In the US, publicly-available ED visit data from the National Hospital Ambulatory Medical Care Survey (NHAMCS) from 1997 through 2007 were assessed using codes from the International Classification of Diseases, Ninth Revision (ICD-9). ED visit rates increased from 352.8 to 390.5 per 1000 persons during that time (*P* = .001 for trend), and adults with Medicaid cover accounted for most of the increase in ED visits; in addition, the visit rate increased from 693.9 to 947.2 visits per 1000 enrollees (*p* = 0.001 for trend) between 1999 and 2007 [14]. According to NHAMCS data from 1997 to 2009, the overall adjusted probability of an ED visit being treatable in a primary care setting increased by 0.19 % (95 % CI = 0.10 to 0.28) per year. This probability increased at a rate of 0.52 % per year for Medicare patient visits (95 % CI = 0.38 % to 0.65 %), more than double that for Medicaid patient visits (0.25 % per year, 95 % CI = 0.13 % to 0.37 %). In contrast, there was no significant change from 1997 to 2009 in the average probability of an ED visit being treatable in a primary care setting in privately-insured patients (0.05 % per year, 95 % CI = −0.07 to 0.16) or uninsured individuals (0.00 % per year, 95 % CI = −0.12 to 0.13) [15]. In Taiwan, the ED visit rate, including traumatic and non-traumatic cases, was approximately 190 visits per 1000 persons in 2001, which increased significantly to 270 visits per 1000 persons in 2010 based on one-million sampled patients who were insured under the official Taiwan health insurance system. Therefore, a greater increase in the ED visit rate as measured by the AAPC, and in particular in the ED visit rate for cases due to traumatic causes, was observed in Taiwan, an Asian developing country.

In the US, older adults (aged 75 and over), non-Hispanic black persons, less wealthy people, and those with Medicaid coverage were more likely to have made at least one ED visit within a 12-month period than other subjects. Persons with Medicaid coverage were more likely to have made multiple visits to an ED within a 12-month period than those with private insurance and the uninsured [16]. Based on data from the USA National Health Interview Survey in 2012, 18 % of children aged 0–17 years visited the ED, and children with Medicaid coverage were more likely than uninsured children and those with private coverage to have visited the ED at least once in the past year [17]. A recent systematic review concluded that in most healthcare systems, frequent ED users were more likely to be older, female, and have a mental health diagnosis, and previous hospitalizations and high primary care use were associated with future frequent ED use [18]. The main finding of the present study was that female subjects and the elderly (aged 65 years or more) exhibited a greater increase in the incidence of ED visits for traumatic reasons in Taiwan.

Overcrowding of EDs is an important issue, and in order to design strategies aimed at avoiding overcrowding, mathematical models used to predict ED patient volume are considered essential. Most of the models used to predict patient volume are linear regression models, including Poisson regression models or time series models that can include calendar- or climate-related variables. These models explain 31–75 % of the patient-volume variability [19–22]. A significantly increased rate of ED utilization was found in the present study, especially with regards to traumatic cases, of which cases of soft tissue trauma of extremities and various fractures were the categories that exhibited the greatest increasing tendencies. This information is useful to inform Taiwan’s health authorities with regards to the delivery and distribution of medical resources.

In Singapore, a statistical report showed that 40.7 % of ambulance arrivals were attributable to trauma, versus 27.3 % of walk-in arrivals. The majority of trauma cases brought in by ambulance was because of road traffic accidents, followed by accidents in the home [23]. In India, traffic crashes and consequent injuries represent a growing public health concern. Open wounds and superficial injuries to the head (69.3 %), upper extremities (27 %) and lower extremities (24 %) were found to be the most common injuries [24]. Based on data from the state-wide trauma registry in Queensland, Australia, from 2006 to 2010, moped/scooter riders sustained a greater percentage of head/neck (+8.6 %), facial (+3.0 %) and abdominal injuries (+2.3 %), whereas motorcycle riders sustained a greater percentage of upper extremity (+4.0 %), thoracic (+3.9 %), spinal (+3.6 %) and lower extremity injuries (+2.6 %) [25]. In Taiwan, the incidence of traffic accident-related hospitalization was between 9.17 and 11.54 %, and in all inpatients admitted due to road traffic accidents, the orthopedic fractures of (1) fracture of upper limb, (2) fracture of lower limb, and (3) fracture of spine/trunk were the most common injuries, accounting for 29.36 % of all injuries [26]. Road traffic accidents involving motorized two-wheeled vehicle (MTV) riders often result in severe morbidity and mortality. Many studies have focused on this specific issue [27, 28]. In the present study, which focused on traumatic injuries, most traumatic cases presenting at EDs had sustained superficial or open-wound injuries; however, the traumatic categories of sprains/strains of joints and adjacent muscles, fractures of upper limbs, fractures of lower limbs, and fractures of the spine/trunk exhibited significant annual increases in incidence. Most traumatic ED cases in the present study were sent to highest-level hospitals, which is contrary to another study performed in Taiwan showing the use of the ED service in the nearest community hospital to be more acceptable in an emergency situation than medical center treatment for dying cancer patients [29]. More detailed evaluation is needed in future studies to examine the causes of accidents and the subsequent morbidity or mortality.

Some limitations were still persisted in the present study. In Taiwan, no researchers can approach all the NHI beneficiaries’ inpatient and ambulatory medical records. A longitudinal health dataset of one million fixed cohort populations, including their inpatient and ambulatory medical records, was the most popular research databank. However, more detailed personal information such as occupation, education level, living area, or healthy habitus were absent. Up to now, there were three isolated longitudinal datasets containing LHID2000, LHID2005 and LHID2010 that can provide only point estimates for epidemiological studies due to lack of available data of whole Taiwan’s population in each calendar year. In another way, during the study period of 2001–2010, 10 separated cross-sectional surveys in each calendar year might be performed based on the truly dynamic total population but only 0.2 % of the ambulatory care records extracted by systematic sampling method have been allowed to be released to Taiwanese researchers [13]. Finally, the latest longitudinal health dataset (LHID2010) in Taiwan was chosen to perform a descriptive epidemiological analysis that was designed as a retrospective fixed-cohort study.

## Conclusions

Focusing on traumatic visits of patients to EDs, increased ED utilization was observed between 2001 and 2010 in Taiwan, as evidenced by the increases in the annual number of cases, annual incidence assessed by gender and age, average direct medical cost, and highest-level hospital utilization. Of the 20 categories into which all traumatic causes were divided, orthopedic-related injuries, including soft tissue trauma of extremities and various fractures, were the categories in which the greatest increases in incidence were observed. Greater numbers of surgical and orthopedic physicians may be needed in EDs, especially in the highest-level hospitals in Taiwan,

## References

[1] O’Reilly GM, Joshipura M, Cameron PA, Gruen R (2013). Trauma registries in developing countries: a review of the published experience. Injury.

[2] Roudsari BS, Nathens AB, Arreola-Risa C, Cameron P, Civil I, Grigoriou G (2007). Emergency Medical Service (EMS) systems in developed and developing countries. Injury.

[3] O’Reilly GM, Gabbe B, Moore L, Cameron PA (2016). Classifying, measuring and improving the quality of data in trauma registries: a review of the literature. Injury.

[4] Man Lo S, Min Yu Y, Larry Lee LY, Eliza Wong ML, Ying Chair S, J Kalinowski E (2012). Overview of the shenzhen emergency medical service call pattern. World J Emerg Med.

[5] Kristiansen T, Lossius HM, Søreide K, Steen PA, Gaarder C, Næss PA (2011). Patients Referred to a Norwegian Trauma Centre: effect of transfer distance on injury patterns, use of resources and outcomes. J Trauma Manag Outcomes.

[6] Kristiansen T, Ringdal KG, Skotheimsvik T, Salthammer HK, Gaarder C, Naess PA (2012). Implementation of recommended trauma system criteria in south-eastern Norway: a cross-sectional hospital survey. Scand J Trauma Resusc Emerg Med.

[7] National Health Insurance Administration, Ministry of Health and Welfare, Taiwan, R.O.C. Background of National Health Insurance Research Database. [http://nhird.nhri.org.tw/en/index.html]. Accessed 1 Aug 2016.

[8] Yang NP, Deng CY, Chou YJ, Chen PQ, Lin CH, Chou P (2006). Estimated prevalence of osteoporosis from a Nationwide Health Insurance database in Taiwan. Health Policy.

[9] Yang NP, Chan CL, Yu IL, Lee CY, Chou P (2010). Estimated prevalence of orthopaedic fractures in Taiwan—a cross-sectional study based on nationwide insurance data. Injury.

[10] Chan CL, Lin W, Yang NP, Huang HT (2013). The association between the availability of ambulatory care and non-emergency treatment in emergency medicine departments: a comprehensive and nationwide validation. Health Policy.

[11] Yang NP, Chan CL, Chu D, Lin JS, Lin KB, Yu CS, et al. Epidemiology of hospitalized traumatic pelvic fractures and their combined injuries in Taiwan: 2000–2011 nationwide surveillance. BioMed Res Int. 2014. Article ID 878601. doi: 10.1155/2014/878601. 10.1155/2014/878601 PMC3988716 24804258

[12] Lin KB, Lai KR, Yang NP, Chan CL, Liu YH, Pan RH (2015). Epidemiology and socioeconomic features of appendicitis in Taiwan: a 12-year population-based study. World J Emerg Surg.

[13] National Health Insurance Administration, Ministry of Health and Welfare, Taiwan, R.O.C. Data subset of National Health Insurance Research Database. [http://nhird.nhri.org.tw/en/Data_Subsets.html]. Accessed 1 Aug 2016.

[14] Tang N, Stein J, Hsia RY, Maselli JH, Gonzales R (2010). Trends and characteristics of US emergency department visits, 1997–2007. JAMA.

[15] Pukurdpol P, Wiler JL, Hsia RY, Ginde AA (2014). Association of Medicare and Medicaid insurance with increasing primary care-treatable emergency department visits in the United States. Acad Emerg Med.

[16] Garcia TC, Bernstein AB, Bush MA (2010). Emergency department visitors and visits: who used the emergency room in 2007?. NCHS Data Brief.

[17] Gindi RM, Jones LI (2014). Reasons for emergency room use among U.S. children: National Health Interview Survey, 2012. NCHS Data Brief.

[18] Soril LJ, Leggett LE, Lorenzetti DL, Noseworthy TW, Clement FM (2016). Characteristics of frequent users of the emergency department in the general adult population: a systematic review of international healthcare systems. Health Policy.

[19] Wargon M, Guidet B, Hoang TD, Hejblum G (2009). A systematic review of models for forecasting the number of emergency department visits. Emerg Med J.

[20] Au-Yeung SW, Harder U, McCoy EJ, Knottenbelt WJ (2009). Predicting patient arrivals to an accident and emergency department. Emerg Med J.

[21] Schweigler LM, Desmond JS, McCarthy ML, Bukowski KJ, Ionides EL, Younger JG (2009). Forecasting models of emergency department crowding. Acad Emerg Med.

[22] McCarthy ML, Zeger SL, Ding R, Aronsky D, Hoot NR, Kelen GD (2008). The challenge of predicting demand for emergency department services. Acad Emerg Med.

[23] Seow E, Wong HP, Phe A (2001). The pattern of ambulance arrivals in the emergency department of an acute care hospital in Singapore. Emerg Med J.

[24] Fitzharris M, Dandona R, Kumar GA, Dandona L (2009). Crash characteristics and patterns of injury among hospitalized motorised two-wheeled vehicle users in urban India. BMC Public Health.

[25] White D, Lang J, Russell G, Tetsworth K, Harvey K, Bellamy N (2013). A comparison of injuries to moped/scooter and motorcycle riders in Queensland, Australia. Injury.

[26] Pan RH, Chang NT, Chu D, Hsu KF, Hsu YN, Hsu JC (2014). Epidemiology of orthopedic fractures and other injuries among inpatients admitted due to traffic accidents: a 10-year nationwide survey in Taiwan. ScientificWorldJournal.

[27] Leijdesdorff HA, Siegerink B, Sier CF, Reurings MC, Schipper IB (2012). Injury pattern, injury severity, and mortality in 33,495 hospital-admitted victims of motorized two-wheeled vehicle crashes in The Netherlands. J Trauma Acute Care Surg.

[28] Miggins M, Lottenberg L, Liu H, Moldawer L, Efron P, Ang D (2011). Mopeds and scooters: crash outcomes in a high traffic state. J Trauma.

[29] Lee YH, Chu D, Yang NP, Chan CL, Cheng SP, Pai JT (2015). Emergency visits among end-of-life cancer patients in Taiwan: a nationwide population-based study. BMC Palliat Care.

